# Multicentre study of the *in vitro* activity of ceftolozane/tazobactam and other commonly used antibiotics against *Pseudomonas aeruginosa* isolates from patients in the UK

**DOI:** 10.1093/jacamr/dlaa024

**Published:** 2020-05-30

**Authors:** Adela Alvarez-Buylla, Mike Allen, Dan Betts, Sean Bennett, Irene Monahan, Tim Planche, INVICTUS study group, Cressida Auckland, Karen Bowker, Helen Chesterfield, Martino Dall’antonia, Mathew Diggle, Noha El Sakka, Wael Elamin, Abid Hussain, Jon Lambourne, John Perry, Timothy Planche, Michael Pryzbylo, Peter Wilson, Mandy Wootton

**Affiliations:** 1 MSD Ltd, Hoddesdon, Hertfordshire, UK; 2 St George’s University Hospitals NHS Foundation Trust, London, UK; 3 Institute of Infection and Immunity, St George’s University of London, London, UK

## Abstract

**Objectives:**

To evaluate the *in vitro* activity of ceftolozane/tazobactam and other commonly used antipseudomonal antibiotics against geographically spread *Pseudomonas aeruginosa* isolates in the UK using disc susceptibility testing.

**Methods:**

The *in vitro* activity of ceftolozane/tazobactam and nine other commonly used antipseudomonal antibiotics was evaluated. Isolates were collected between January 2015 and April 2018. Susceptibility results were interpreted using EUCAST 2018 criteria.

**Results:**

Overall, 1326 clinical isolates from 14 centres in the UK were tested. The majority of the isolates were collected from non-cystic fibrosis (non-CF) patients (*n *=* *1123, 85.0%). In addition, 199 cystic fibrosis (CF) isolates were collected from 10 centres. Overall susceptibility to ceftolozane/tazobactam was 89.3% (*n *=* *1181), which included 128 CF and 1053 non-CF isolates. The other antibacterial agents with the highest susceptibility were tobramycin (92.4%, *n *=* *1221) and piperacillin/tazobactam (90.7%, *n *=* *1199). Susceptibility to all antibacterial agents was lower for CF isolates. Piperacillin/tazobactam was the most active of the antibacterial agents tested, followed by ceftolozane/tazobactam (70.4% and 64.3%, respectively), and <60% of CF isolates were susceptible to ceftazidime and the carbapenems. The reason for the higher rates of susceptibility to piperacillin/tazobactam and lower susceptibility to ceftazidime compared with other studies is unclear.

**Conclusions:**

The data presented here support the need to investigate the place of ceftolozane/tazobactam as a treatment option in the management of pseudomonal infections, particularly in patients with CF. The results highlight the importance of routine testing of new antibacterial agents and of making the data available to clinicians to make appropriate and informed treatment choices.

## Introduction

*Pseudomonas aeruginosa* is an important pathogen of healthcare-associated infections, including nosocomial pneumonia and urinary tract and bloodstream infections. In a point prevalence study conducted in ICUs in Western Europe, *P. aeruginosa* was one of the most commonly reported pathogens, constituting 29% of all Gram-negative isolates and present in 17% of all positive cultures.^1^*P. aeruginosa* is also a clinically important, opportunistic pathogen in patients with cystic fibrosis (CF), with infections often establishing a pattern of persistence and strains undergoing a phenotypic change characterized by the production of the polysaccharide alginate.^2^ It has been estimated that more than 80% of CF patients will succumb to respiratory failure caused by chronic bacterial infection and concomitant airway inflammation.^3^ The intrinsic antibiotic resistance of *P. aeruginosa* and reported levels of acquired resistance, including MDR strains, highlight the need for both surveillance and novel antibacterial agents for the treatment of these infections. The ECDC 2018 report documented that of the 16 885 invasive isolates of *P. aeruginosa* tested, 5201 (30.8%) were resistant to a single antimicrobial group and 659 (3.9%) were resistant to all five groups.^4^ A global priority pathogen list (PPL) ranking the bacteria that pose the greatest threat to human health highlighted, in particular, Gram-negative bacteria that are resistant to multiple antibiotics as high risk and identified carbapenem-resistant (CR) *P. aeruginosa* as a critical organism.^5^ These levels of resistance and MDR trends present a therapeutic challenge, particularly in patients receiving treatment on ICUs or with comorbidities.^6^

Ceftolozane/tazobactam is a novel β-lactam/β-lactamase inhibitor combination with potent activity against Gram-negative bacteria, particularly against *P. aeruginosa*, for which it is the most active β-lactam antibiotic available.^7^ Ceftolozane/tazobactam has been shown to be non-inferior to comparators in Phase III trials investigating complicated intra-abdominal infection (cIAI), complicated urinary tract infection (cUTI) and hospital-acquired pneumonia (HAP), including ventilator-associated pneumonia (VAP),^8–10^ with excellent *in vitro* activity against MDR and XDR *Pseudomonas* spp.,^6^ including strains with derepressed AmpC or up-regulated efflux.^11^ BSAC data in the UK report ceftolozane/tazobactam as a potent antipseudomonal antibiotic *in vitro*, with higher susceptibility rates than other β-lactam/β-lactamase inhibitor combinations, carbapenems and fluoroquinolones. Susceptibility rates have been consistently high over the 7 years analysed (2011–17), with 99.5%, 99.5%, 100%, 100%, 100%, 99.4% and 99.5% bacteraemia isolates susceptible to ceftolozane/tazobactam, respectively.^12^ For respiratory isolates, susceptibility rates to ceftolozane/tazobactam were 98.1% for the 2015–16 period and 100% for the 2016–17 period.^13^

Data on the activity of ceftolozane/tazobactam against *P. aeruginosa* were limited in the licensing trials. Despite the increasing number of publications of *in vitro* studies,^7^^,^^14–21^ national and local data are needed to guide informed prescribing and identify resistance trends, treatment strategies and laboratory protocols.

Disc susceptibility testing is a well-established, easy-to-perform and inexpensive methodology used in many laboratories as a first-line test. Disc testing offers practical advantages over other antimicrobial susceptibility testing methods for ceftolozane/tazobactam, such as broth dilution or MIC gradient strips. It is versatile in the range of antimicrobial agents that can be tested, requires no special equipment and is inexpensive. Disc diffusion may be the sole antimicrobial susceptibility testing method in diagnostic microbiology laboratories where no automated alternatives are available.

The purpose of the current study was to evaluate the *in vitro* activity of ceftolozane/tazobactam and other commonly used antipseudomonal antibiotics against geographically spread *P. aeruginosa* isolates in the UK. A secondary objective was to provide centres with the opportunity to generate local susceptibility data to guide appropriate antipseudomonal therapy.

## Methods

### Study setting and design

A multicentre, real-world study of clinically significant consecutive isolates of *P. aeruginosa* collected across England, Scotland and Wales, including isolates from CF patients, was conducted. A total of 14 diagnostic laboratories were asked to collect 100 consecutive *P. aeruginosa* isolates that were deemed clinically relevant from samples submitted to the laboratory over a 2 month period. If insufficient isolates were collected, then laboratories could complete the 100 clinically relevant isolates from isolates stored for clinical reasons. Isolates from the same patient were excluded from the study.

### Antimicrobial identification and susceptibility testing

MALDI-TOF MS methodology was used for species identification in the local diagnostic laboratory. *In vitro* susceptibility testing following the EUCAST disc diffusion methodology, with discs obtained from MAST Group Ltd, was used to assess the susceptibility of *P. aeruginosa* isolates to ceftolozane/tazobactam and nine other antipseudomonal antibiotics (piperacillin/tazobactam, ceftazidime, imipenem, meropenem, aztreonam, amikacin, gentamicin, tobramycin and ciprofloxacin). Mueller–Hinton agar was the medium used for the disc diffusion methodology, as recommended by EUCAST. Sites were allowed to use their preferred manufacturer. Disc diffusion was selected as the preferred methodology for the study because of its widespread use, convenience and reliability.^22^ Susceptibility results were interpreted using EUCAST 2018 criteria.^23^

*P. aeruginosa* (ATCC 27853) and either *Escherichia coli* (ATCC 35218) or *Klebsiella pneumoniae* (ATCC 700603) were used as the quality control strains at every centre to monitor the performance of the tests. These control strains were tested daily whenever study isolates were analysed. Internal quality control failure invalidated the results and required repeat analysis.

The susceptibility of at least 10% of the isolates per centre was reassessed at the Central Testing Laboratory for reproducibility and external quality control on an isolate number exactly divisible by 10, in line with United Kingdom Accreditation Service (UKAS) guidance. In addition, all isolates with a zone diameter for ceftolozane/tazobactam within ±1 mm of the breakpoint (i.e. 23–25 mm), as per each referring centre’s susceptibility testing results, was included in the robust external quality control study. All testing in the Central Testing Laboratory was performed blind to previous results and testing was performed in triplicate per isolate.

Isolates identified at the local laboratories using a methodology other than MALDI-TOF MS (Bruker, Germany) were reidentified by MALDI-TOF MS at the Central Testing Laboratory.

### Statistical methods and analysis

The number of participating centres was made to reflect the BSAC Resistance Surveillance Programme^24^ and provide a good geographical spread across the UK. Consecutive isolates from each centre were required by the protocol in order to reduce the risk of centres submitting isolates from particular patients or sample types. A cap of 100 isolates, with no more than 50% from CF patients per centre, was used to minimize sample size variability across participant centres. No minimum number of CF isolates was required from each centre.

Data were collected in an Excel spreadsheet with inhibition zone diameter (mm) range limit for each antibiotic inhibition zone. A minimal dataset of the following was collected for each isolate: date of sample, whether the sample was from a CF patient, whether the isolate was phenotypically mucoid or non-mucoid, the source of the sample, the presumed source of the infection and the specialty sending the sample. Centres were provided with a list of sample sources, likely sites of infection and specialties.

The *in vitro* activity of ceftolozane/tazobactam and the nine other antibacterial agents was expressed as inhibition zone diameter (mm) and summarized for all isolates and by centre. The original zone size quoted by the diagnostic laboratory was used in the analysis. Further analysis on the basis of retesting isolates in the Central Testing Laboratory was based on a mean of the zone diameters of the original and three repeat measures. Percentages of susceptible and resistant isolates per antibiotic were calculated using EUCAST breakpoints^23^ and further analysed by antibiotic, ward, sample source and CF/non-CF (patients without CF) isolates.

### Ethics

Only bacterial isolates were included and analysed in the study. No human tissue was collected, stored or analysed and no patient data were recorded, thus ethics approval was not applicable for this study. Health Research Authority (HRA) approval was obtained.

## Results

A total of 14 centres participated in the study, with the majority being tertiary or teaching centres with large ICUs and CF units. Isolates were collected from specimens submitted to the laboratories between January 2015 and April 2018.

A total of 1326 isolates were included in the study. Twelve of the sites contributed 100 isolates each. The remaining two sites tested 69 and 57 isolates. MALDI-TOF MS identification confirmed 1322 (1123 non-CF and 199 CF) as *P. aeruginosa*. Other species were *Pseudomonas otitidis* (*n *=* *2), *Pseudomonas corrugata* (*n *=* *1) and *Pseudomonas mosselii* (*n *=* *1). The *in vitro* activities of ceftolozane/tazobactam and nine comparators are summarized in Table 1 for all of the 1322 *P. aeruginosa* isolates collected from these 14 UK sites.

**Table 1.** Susceptibility of *P. aeruginosa* isolates, including those from patients with CF

	C/T	TZP	CAZ	IPM	MEM	ATM	AMK	GEN	TOB	CIP
Overall (*n *=* *1322)										
SUS, *n*	1181	1199	1051	1090	1050	0	1145	1121	1221	973
%	89.3	90.7	79.5	82.5	79.4	0	86.6	84.8	92.4	73.6
INT, *n*	—	—	—	38	81	1136	73	—	—	—
%				2.9	6.1	85.9	5.5			
RES, *n*	141	123	271	194	191	186	104	201	101	349
%	10.7	9.3	20.5	14.7	14.4	14.1	7.9	15.2	7.6	26.4
CF (*n *=* *199)										
SUS, *n*	128	140	95	105	105	0	97	103	132	70
%	64.3	70.4	47.7	52.8	52.8		48.7	51.8	66.3	35.2
INT, *n*	—	—	—	7	13	129	23	—	—	—
%				3.5	6.5	64.8	11.6			
RES, *n*	71	59	104	87	81	70	79	96	67	129
%	35.7	29.6	52.3	43.7	40.7	35.2	39.7	48.2	33.7	64.8
Non-CF (*n *=* *1123)										
SUS, *n*	1053	1059	956	985	945	0	1048	1018	1089	903
%	93.8	94.3	85.1	87.7	84.1		93.3	90.7	97.0	80.4
INT, *n*	—	—	—	31	68	1007	50	—	—	—
%				2.8	6.5	89.7	4.5			
RES, *n*	70	64	167	107	110	116	25	105	34	220
%	6.2	5.7	14.9	9.5	9.8	10.3	2.2	9.3	3.0	19.6

— indicates that there is no gap between susceptible and resistant zone diameters.

C/T, ceftolozane/tazobactam; TZP, piperacillin/tazobactam; CAZ, ceftazidime; IPM, imipenem; MEM, meropenem; ATM, aztreonam; AMK, amikacin; GEN, gentamicin; TOB, tobramycin; CIP, ciprofloxacin; INT, intermediate; RES, resistant; SUS, susceptible.

**Figure 1.**
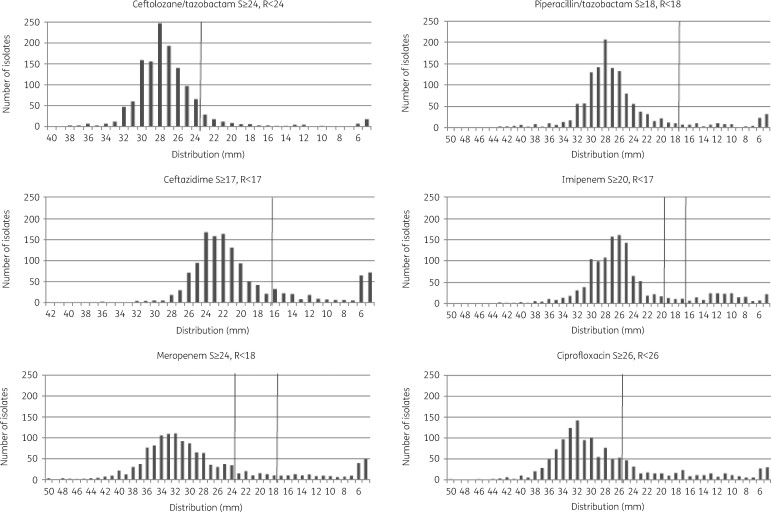
Distribution of zone diameters (mm) for all 1322 *P. aeruginosa* isolates to ceftolozane/tazobactam, piperacillin/tazobactam, ceftazidime, meropenem, imipenem and ciprofloxacin. EUCAST 2018 breakpoints are included for each antibiotic and marked on the figure.

Of the isolates tested, 89.3% (*n *=* *1181) were susceptible to ceftolozane/tazobactam, which included 128 CF and 1053 non-CF isolates. The three antipseudomonals with the highest percentage susceptibility were tobramycin (92.4%, *n *=* *1221), piperacillin/tazobactam (90.7%, *n *=* *1199) and ceftolozane/tazobactam (89.3%, *n *=* *1181). Figure 1 shows the distribution of zone diameters (mm) for all 1322 *P. aeruginosa* isolates to ceftolozane/tazobactam, piperacillin/tazobactam, ceftazidime and meropenem. The majority of other antibacterial agents had lower susceptibility rates and the single fluoroquinolone tested in this study, ciprofloxacin, had an overall susceptibility rate of 73.6% (*n *=* *973).

### Retesting in Central Testing Laboratory and reproducibility

The three antipseudomonals with the highest percentage susceptibility after retesting in the Central Testing Laboratory were again tobramycin (92.7%, *n *=* *1226), ceftolozane/tazobactam (91.5%, *n *=* *1209) and piperacillin/tazobactam (90.8%, *n *=* *1200) and there were no statistical differences in susceptibility to these three antibacterial agents. The reproducibility of the disc diameter measurements varied between antibacterial agents tested (Table 2). Ceftolozane/tazobactam had the lowest coefficient of variation (4.8%) for disc diameter, along with imipenem (6.8%), amikacin (7.3%), tobramycin (5.3%) and ciprofloxacin (6.9%).

**Table 2.** Coefficient of variation of zone size diameter as retested three times by Central Testing Laboratory

	C/T	TZP	CAZ	IPM	MEM	ATM	AMK	GEN	TOB	CIP
*N*	143	143	143	143	143	143	143	143	143	143
Coefficient of variation (%)	4.8	10.3	27.0	6.8	17.1	15.6	7.8	9.4	5.3	6.9

C/T, ceftolozane/tazobactam; TZP, piperacillin/tazobactam; CAZ, ceftazidime; IPM, imipenem; MEM, meropenem; ATM, aztreonam; AMK, amikacin; GEN, gentamicin; TOB, tobramycin; CIP, ciprofloxacin.

### Isolates from non-CF and CF patients

The majority of the total isolates were collected from non-CF patients (*n *=* *1123, 85.0%) and susceptibility was consistent with the overall results. Tobramycin, piperacillin/tazobactam and ceftolozane/tazobactam were the three most active antibacterial agents against all non-CF isolates, with susceptibilities of 97.0%, 94.3% and 93.8%, respectively. In line with the overall results, no isolates were susceptible to aztreonam (Figure S1, available as Supplementary data at *JAC-AMR* Online).

A total of 199 CF isolates were collected from 10 centres, with 148 isolates (74.4%) originating from respiratory wards and the remaining isolates obtained from a ward unknown to the investigators. Sputum was the primary sample source (*n *=* *176, 88.4%), with the most likely site of infection being the lower respiratory tract (*n *=* *195, 98.0%). Overall, susceptibility to all antibacterial agents was lower in comparison with non-CF isolates (Table 1). Piperacillin/tazobactam was the most active of the antibacterial agents tested, followed by ceftolozane/tazobactam (70.4% and 64.3%, respectively). Over 40% of CF isolates were resistant to ceftazidime and the carbapenems (ceftazidime 52.3%, imipenem 43.7% and meropenem 40.7%). Tobramycin was the only aminoglycoside that showed *in vitro* activity >55%, with susceptibility rates of 66.3% (Figure S2).

### Type of ward

Isolates were collected from multiple wards at each centre, with the majority coming from respiratory wards (*n *=* *220, 16.6%), ICUs (*n *=* *151, 11.4%) and acute medical wards (*n *=* *144, 10.9%) (Table S1). Susceptibility rates for ceftolozane/tazobactam ranged from 71.8% (respiratory wards, *n *=* *220) to 100% (nephrology wards, *n *=* *33). Isolates collected from respiratory wards showed the lowest susceptibility to all antibacterial agents but were generally more susceptible to β-lactams (58.2% to 76.0%), gentamicin (61.4%) and tobramycin (74.1%). Aminoglycosides were the most active class against isolates collected from ICUs (tobramycin 94.0%, amikacin 94.7% and gentamicin 91.4%, respectively).

### Sample source

Samples were collected from various sources [sputum (*n *=* *420), blood (*n *=* *251), wound swab (*n *=* *235), urine (*n *=* *204), pus (*n *=* *31), bronchoalveolar lavage (BAL) (*n *=* *20), CSF (*n *=* *5) and other (*n *=* *156)]. Rates of susceptibility to ceftolozane/tazobactam of isolates from blood, wound swab and urine samples were 96.8%, 95.3% and 91.7%, respectively. Susceptibility of all sputum isolates, including those from patients with CF, was 79.3%. Of the other β-lactams tested, piperacillin/tazobactam was the most active across all sample sources and susceptibility ranged from 82.9% for sputum samples to 100% for CSF samples. The susceptibility to ceftazidime and the carbapenems was lower compared with ceftolozane/tazobactam and piperacillin/tazobactam across all sample sources. More than 90% of isolates from blood cultures, pus and wound swabs were reported as susceptible to the three aminoglycosides tested. As with β-lactams, susceptibility rates to aminoglycosides in sputum were generally lower (amikacin 72.1%, gentamicin 72.9% and tobramycin 83.6%). The monobactam aztreonam and the fluoroquinolone ciprofloxacin were the least active antibacterial agents across all sample sources (Table 3).

**Table 3.** Susceptibility of isolates to all antibacterial agents per sample source (%) (*n *=* *1322)

Sample source	*N*	C/T		TZP		CAZ		IPM			MEM			ATM			AMK			GEN		TOB		CIP	
		S%	R%	S%	R%	S%	R%	S%	I%	R%	S%	I%	R%	S%	I%	R%	S%	I%	R%	S%	R%	S%	R%	S%	R%
Blood	251	96.8	3.3	94.0	6.0	82.9	17.1	88.8	4.0	7.2	84.5	7.6	8.0	0	90.4	9.6	96.0	1.6	2.4	93.2	6.8	94.4	5.6	81.7	18.3
BAL	20	80.0	25.0	85.0	15.0	85.0	15.0	85.0	0	15.0	80.0	5.0	15.0	0	85.0	15.0	95.0	0	5.0	90.0	10.0	95.0	5.0	95.0	5.0
Sputum	420	79.3	26.1	82.9	17.1	66.7	33.3	71.0	2.9	26.2	68.3	6.4	25.2	0	77.9	22.1	72.1	8.1	19.8	72.9	27.1	83.6	16.4	57.4	42.6
CSF	5	100	0	100	0	100	0	80.0	0	20.0	80.0	0	20.0	0	80.0	20.0	100	0	0	100	0	100	0	100	0
Urine	204	91.7	9.1	96.1	3.9	83.3	16.7	89.7	3.4	6.9	86.3	5.9	7.8	0	89.7	10.3	86.3	11.8	2.0	82.8	17.2	97.5	2.5	75.0	25.0
Pus	31	83.9	19.2	93.5	6.5	83.9	16.1	93.5	3.2	3.2	90.3	6.5	3.2	0	87.1	12.9	100	0	0	100	0	100	0	90.3	9.7
Wound swab	235	95.3	4.9	94.0	6.0	89.4	10.6	86.4	3.0	10.6	84.3	4.3	11.5	0	90.2	9.8	95.3	3.4	1.3	91.9	8.1	97.0	3.0	83.8	16.2
Other	156	94.2	6.1	94.2	5.8	86.5	13.5	85.3	0.6	14.1	82.7	6.4	10.9	0	89.1	10.9	93.6	1.9	4.5	91.0	9.0	96.8	3.2	80.1	19.9

C/T, ceftolozane/tazobactam; TZP, piperacillin/tazobactam; CAZ, ceftazidime; IPM, imipenem; MEM, meropenem; ATM, aztreonam; AMK, amikacin; GEN, gentamicin; TOB, tobramycin; CIP, ciprofloxacin; I, intermediate; R, resistant; S, susceptible.

### Site of infection

Sites of infection from which isolates were collected included lower respiratory tract (*n *=* *481, 36.4%), urinary tract (*n *=* *245, 18.5%), skin and soft tissue (*n *=* *214, 16.2%), intra-abdominal (*n *=* *60, 4.5%), burns (*n *=* *22, 1.7%), prosthetic joints (*n *=* *17, 1.3%), CNS (*n *=* *5, 0.4%) and other sites (*n *=* *278, 21.0%). Susceptibility to ceftolozane/tazobactam ranged between 80.9%, for lower respiratory tract infections, to 100% for CNS; however, CNS isolates were very limited (*n *=* *5). Susceptibility to ceftolozane/tazobactam in UTI isolates was high (92.2%) but lower than in skin and soft tissue infections (96.3%). Isolates from lower respiratory tract infections had the lowest rates of susceptibility to all antibacterial agents tested, with values ranging from 60.7% for ciprofloxacin to 84.8% for tobramycin. (Details of the sites of infection and susceptibilities to the other antibacterial agents tested can be found in Table S2).

## Discussion

*P. aeruginosa* is ubiquitous in aquatic environments and difficult to eradicate because of both its versatility and intrinsic tolerance to many detergents, disinfectants and antimicrobial agents.^25^ In addition, increasing levels of resistance and MDR strains, particularly in ICUs, highlights the need for new, effective agents for treatment.^26^ This is a large comparative study showing the susceptibility to ceftolozane/tazobactam and commonly used antipseudomonal antibiotics across the UK.

The findings reported here confirm the activity of ceftolozane/tazobactam, with an overall susceptibility rate of 89.3% and a susceptibility rate of 88.1% for isolates obtained from ICUs. Most of the data are consistent with proportions of isolates susceptible to ceftolozane/tazobactam reported elsewhere. For example, the bacteraemia data for ceftolozane/tazobactam in this study (97% of blood isolates being susceptible to ceftolozane/tazobactam) are consistent with data from the BSAC Resistance Surveillance Programme and UK national reference laboratory surveillance data collected between 2011 and 2017, which reported 99.4% to 100% of isolates as susceptible to ceftolozane/tazobactam.^12^

In the current study, of the 20 isolates obtained from BAL samples and the 420 from sputum, susceptibility to ceftolozane/tazobactam was 80.0% and 79.3%, respectively. These susceptibility rates are lower than those reported by BSAC for the periods 2015–16 (98.1%) and 2016–17 (100%), respectively,^13^ although it should be noted that the BSAC Resistance Surveillance Programme excludes isolates from CF patients.

Susceptibility rates for other commonly used antibacterial agents for the treatment of pseudomonal infections were also high; ceftolozane/tazobactam remained more active *in vitro* in the BSAC study than other β-lactams, β-lactam/β-lactamase inhibitor combinations, carbapenems, aminoglycosides and fluoroquinolones. Susceptibility to ceftolozane/tazobactam of sputum isolates was lower in the current study (79.3%). However, 42% of these isolates came from CF patients, where susceptibility rates to all antibacterial agents were predictably lower. The high percentage susceptibility to ceftolozane/tazobactam and tobramycin was anticipated, although piperacillin/tazobactam susceptibility rates were higher than those reported in some other studies, including the BSAC Resistance Surveillance Programme.^13^ Interestingly, recent data from the BSAC Resistance Surveillance Programme showed a decline in the rate of resistance to piperacillin/tazobactam, with 97.6% of *P. aeruginosa* isolates from blood in 2017 and 90.6% of respiratory isolates collected between 2016 and 2017 being susceptible to piperacillin/tazobactam (http://www.bsacsurv.org/reports/bacteraemia#results). However, bacteraemia data from PHE reported a decline in susceptibility to piperacillin/tazobactam from 91% in 2015 to 86% in 2017^27^ and further longitudinal data are needed to interpret the significance of these conflicting trends. Sader *et al.*^28^ reported overall susceptibility of *Pseudomonas* spp. to piperacillin/tazobactam in the USA in 2017 as 77.5% and an Australian study of blood isolates reported an overall susceptibility of 67% for blood isolates collected over a 10 year period (2008 to 2018).^29^ It is important to consider that it is difficult to compare the findings of the current study, which used disc diffusion methodology, with those of international studies that employed MIC CLSI methodology.

Unexpectedly low susceptibility rates to ceftazidime were seen in the current study (overall 79.5%; non-CF isolates 85.1%) when compared with those reported by BSAC (98.1% for the 2017 bacteraemia isolates and 94.6% for the 2016–17 respiratory isolates). This observation was particularly evident for the blood isolates, where the susceptibility rate for ceftazidime in the current study was 82.9%. The 2017 data from PHE bacteraemia isolates also reported higher susceptibility rates of 93% for ceftazidime.^27^ In the current study, ceftazidime susceptibility for respiratory isolates was 68.2%, compared with 94.6% for the 2016–17 BSAC respiratory isolates. The latter finding may, in part, be explained by the inclusion of CF isolates in the respiratory samples collected in the study.

The recent introduction by EUCAST of an area of technical uncertainty (ATU) of 18–19 mm for piperacillin/tazobactam,^30^ where interpretation of results from disc diffusion is uncertain, may help explain the differences reported in the current study, where use of the 2018 EUCAST criteria^23^ may have overestimated the number of susceptible isolates. Although this clearly warrants further investigation, it is outside the scope of the current study. The 2015 data from the EARSS surveillance database reported a range of susceptibilities to piperacillin/tazobactam, from 100% (Iceland and Luxembourg) to 43% (Romania).^25^ Data from the USA collected between 2012 and 2015 reported overall susceptibilities of 97% to ceftolozane/tazobactam and 80% to piperacillin/tazobactam.^7^ Nearly half of the CF isolates were non-susceptible to ceftazidime and the carbapenems (ceftazidime 52.3%, imipenem 47.2% and meropenem 47.2%). Tobramycin was the only aminoglycoside that showed *in vitro* activity above 55%, with susceptibility rates (66.3%) similar to those of ceftolozane/tazobactam. Of all the antibacterial agents included in the study, tobramycin had the highest *in vitro* susceptibility rates for CF and non-CF isolates: 66.3% and 97.0%, respectively.

As stated previously, the reason for the higher rates of susceptibility to piperacillin/tazobactam and lower susceptibility to ceftazidime recorded in the current study compared with other studies is unclear and further investigation is needed, especially as ceftazidime remains one of the recommended treatments for the management of pseudomonal infections, as described in the Joint Working Party (JWP) MDR Gram-negative guidelines.^31^ The JWP guidelines highlight ceftazidime resistance as pivotal in demonstrating the need for new antibacterial agents, such as ceftolozane/tazobactam, rather than the WHO position, which identifies CR *P. aeruginosa*, because most resistance to carbapenems is characterized by porin loss and efflux mechanisms.^32^

The use of disc susceptibility testing is confirmed as useful in this study, given the coefficients of variation of zone size diameter seen, with the exception of ceftazidime. The absolute necessity for local susceptibility data and the cheapness, speed and simplicity of disc testing further support its use.

Limitations of the study are that there may have been variation in which isolates were considered clinically significant, though it seems unlikely this would account for the differences in susceptibilities reported here. For practicality, disc susceptibility testing was used for the samples, whereas broth dilution is the reference standard for determination of antibiotic susceptibility, particularly as colistin susceptibility cannot be tested with disc methodology. The already noted reproducibility of disc zone diameters and agreement with previously reported results is supportive of using disc diffusion methodology.

Although ceftolozane/tazobactam has been approved only for the treatment of cUTI, cIAI and HAP/VAP, it has become a suitable and attractive option for the treatment of MDR or XDR *P. aeruginosa*.^33^ A prospective observational study of 58 patients receiving ceftolozane/tazobactam monotherapy (*n *=* *21, 36.2%) or combination therapy for ≥72 h (*n *=* *37, 63.8%) reported a clinical cure in 37 patients (63.8%) and resistance development in 8 (13.8%).^34^ Other recent reports include the clinical use of ceftolozane/tazobactam in patients with osteomyelitis and skin and soft tissue infections^35^ and haematologic malignancies and transplant recipients.^36^

The data presented here support the need to investigate the place of ceftolozane/tazobactam as an effective treatment option in the management of pseudomonal infections, particularly in patients with CF. In addition, further studies are needed to address the omission of colistin from the current study. The results presented here highlight the importance of routine testing of new antibacterial agents and of making the data available to clinicians to make appropriate and informed treatment choices.

## Supplementary Material

dlaa024_Supplementary_Data
