# Supplementary material for: Multicentre study of the in vitro activity of ceftolozane/tazobactam and other commonly used antibiotics against Pseudomonas aeruginosa isolates from patients in the UK
Source: JAC Antimicrob Resist. 2020 May 30;2(2):dlaa024. doi: 10.1093/jacamr/dlaa024 (PMC8209992; doi:10.1093/jacamr/dlaa024)
Supplement: dlaa024_Supplementary_Data [file dlaa024_supplementary_data.zip › Supplementary_data.docx]

**Supplementary data**

Table S1. Susceptibility of isolates to all antibacterial agents per ward (%) (N=1,322)

|  |  | **C/T** | | **TZP** | | **CAZ** | | **IPM** | | | **MEM** | | | **ATM** | | | **AMK** | | | **GEN** | | **TOB** | | **CIP** | |  |
| --- | --- | --- | --- | --- | --- | --- | --- | --- | --- | --- | --- | --- | --- | --- | --- | --- | --- | --- | --- | --- | --- | --- | --- | --- | --- | --- |
| **Ward** | n | S% | R% | S% | R% | S% | R% | S% | I% | R% | S% | I% | R% | S% | I% | R% | S% | I% | R% | S% | R% | S% | R% | S% | R% | |
| ICU | 151 | 88.1 | 13.5 | 82.8 | 17.2 | 75.5 | 24.5 | 78.8 | 3.3 | 17.9 | 70.9 | 7.3 | 21.9 | 0 | 79.5 | 20.5 | 94.7 | 2.0 | 3.3 | 91.4 | 8.6 | 94.0 | 6.0 | 72.2 | 27.8 | |
| RESP | 220 | 71.8 | 39.2 | 75.9 | 24.1 | 58.2 | 41.8 | 60.9 | 3.2 | 35.9 | 60.5 | 5.5 | 34.1 | 0 | 73.2 | 26.8 | 58.6 | 10.9 | 30.5 | 61.4 | 38.6 | 74.1 | 25.9 | 44.5 | 55.5 | |
| ED | 108 | 93.5 | 6.9 | 96.3 | 3.7 | 86.1 | 13.9 | 90.7 | 2.8 | 6.5 | 86.1 | 7.4 | 6.5 | 0 | 92.6 | 7.4 | 94.4 | 3.7 | 1.9 | 93.5 | 6.5 | 97.2 | 2.8 | 84.3 | 15.7 | |
| GAS | 17 | 88.2 | 13.3 | 100 | 0 | 82.4 | 17.6 | 82.4 | 5.9 | 11.8 | 82.4 | 5.9 | 11.8 | 0 | 82.4 | 17.6 | 94.1 | 0 | 5.9 | 88.2 | 11.8 | 94.1 | 5.9 | 58.8 | 41.2 | |
| SURG | 118 | 94.1 | 6.3 | 93.2 | 6.8 | 89.8 | 10.2 | 89.0 | 3.4 | 7.6 | 85.6 | 2.5 | 11.9 | 0 | 90.7 | 9.3 | 89.8 | 7.6 | 2.5 | 88.1 | 11.9 | 98.3 | 1.7 | 87.3 | 12.7 | |
| NEPH | 33 | 100 | 0 | 97.0 | 3.0 | 72.7 | 27.3 | 93.9 | 0.0 | 6.1 | 93.9 | 3.0 | 3.0 | 0 | 87.9 | 12.1 | 90.9 | 9.1 | 0 | 87.9 | 12.1 | 97.0 | 3.0 | 72.7 | 27.3 | |
| AM | 144 | 93.1 | 7.5 | 93.1 | 6.9 | 77.8 | 22.2 | 88.9 | 4.2 | 6.9 | 84.0 | 9.0 | 6.9 | 0 | 88.2 | 11.8 | 96.5 | 2.8 | 0.7 | 95.8 | 4.2 | 98.6 | 1.4 | 84.0 | 16.0 | |
| Other | 531 | 93.4 | 7.1 | 96.0 | 4.0 | 86.6 | 13.4 | 86.8 | 2.3 | 10.9 | 84.7 | 6.0 | 9.2 | 0 | 90.0 | 10.0 | 90.4 | 4.9 | 4.7 | 86.8 | 13.2 | 95.1 | 4.9 | 78.5 | 21.5 | |

C/T, ceftolozane/tazobactam; TZP, piperacillin/tazobactam; CAZ, ceftazidime; IPM, imipenem; MEM, meropenem; ATM, aztreonam; AMK, amikacin; GEN, gentamicin; TOB, tobramycin; CIP, ciprofloxacin

ICU= intensive care unit, RESP= respiratory, ED= emergency department, GAS=gastroenterology, SURG= general surgery, NEPH= Nephrology, AM= acute medicine

Table S2. Susceptibility of isolates to all antibacterial agents per site of infection (%) (N=1,322).

|  |  | C/T | | TZP | | CAZ | | IPM | | | MEM | | | ATM | | | AMK | | | GEN | | TOB | | CIP | |
| --- | --- | --- | --- | --- | --- | --- | --- | --- | --- | --- | --- | --- | --- | --- | --- | --- | --- | --- | --- | --- | --- | --- | --- | --- | --- |
| **Likely site of infection** | n | S% | R% | S% | R% | S% | R% | S% | I% | R% | S% | I% | R% | S% | I% | R% | S% | I% | R% | S% | R% | S% | R% | S% | R% |
| LRTI | 481 | 80.9 | 23.7 | 83.4 | 16.6 | 68.2 | 31.8 | 72.8 | 2.9 | 24.3 | 70.3 | 6.4 | 23.3 | 0 | 78.6 | 21.4 | 74.0 | 7.7 | 18.3 | 74.4 | 25.6 | 84.8 | 15.2 | 60.7 | 39.3 |
| UT | 245 | 92.2 | 8.4 | 96.3 | 3.7 | 82.4 | 17.6 | 89.4 | 3.7 | 6.9 | 85.7 | 6.1 | 8.2 | 0 | 90.2 | 9.8 | 87.8 | 10.2 | 2.0 | 84.1 | 15.9 | 96.7 | 3.3 | 75.1 | 24.9 |
| IAI | 60 | 85.0 | 17.6 | 88.3 | 11.7 | 75.0 | 25.0 | 88.3 | 5.0 | 6.7 | 81.7 | 8.3 | 10.0 | 0 | 80.0 | 20.0 | 96.7 | 0 | 3.3 | 96.7 | 3.3 | 96.7 | 3.3 | 68.3 | 31.7 |
| CNS | 5 | 100 | 0 | 80.0 | 20.0 | 80.0 | 20.0 | 100 | 0 | 0 | 100 | 0 | 0 | 0 | 80.0 | 20.0 | 100 | 0 | 0 | 100 | 0 | 100 | 0 | 100 | 0 |
| SSTI | 214 | 96.3 | 3.9 | 96.3 | 3.7 | 91.1 | 8.9 | 87.9 | 2.8 | 9.3 | 86.9 | 4.7 | 8.4 | 0 | 93.0 | 7.0 | 98.1 | 0.9 | 0.9 | 95.8 | 4.2 | 98.1 | 1.9 | 85.0 | 15.0 |
| PJI | 17 | 94.1 | 6.3 | 94.1 | 5.9 | 88.2 | 11.8 | 94.1 | 0.0 | 5.9 | 88.2 | 11.8 | 0.0 | 0 | 88.2 | 11.8 | 100 | 0 | 0 | 100 | 0 | 100 | 0 | 100 | 0 |
| BI | 22 | 90.9 | 10.0 | 81.8 | 18.2 | 77.3 | 22.7 | 77.3 | 9.1 | 13.6 | 59.1 | 18.2 | 22.7 | 0 | 81.8 | 18.2 | 86.4 | 13.6 | 0 | 86.4 | 13.6 | 95.5 | 4.5 | 63.6 | 36.4 |
| Other | 278 | 96.4 | 3.7 | 95.3 | 4.7 | 88.1 | 11.9 | 87.1 | 1.4 | 11.5 | 84.2 | 5.0 | 10.8 | 0 | 91.0 | 9.0 | 95.3 | 2.2 | 2.5 | 91.0 | 9.0 | 95.3 | 4.7 | 85.6 | 14.4 |

LRTI=Lower respiratory tract infection, UTI= Urinary tract infection, IAI= infection, infection, CNS= central nervous system infection, SSTI= skin and soft tissue infection, PJI= prosthetic joint infection, BI= burn infection.

**Figure S1**

Figure S1. Overall susceptibility of all non-CF isolates (%), irrespective of source and site of infection (N=1,123)

C/T, ceftolozane/tazobactam; TZP, piperacillin/tazobactam; CAZ, ceftazidime; IPM, imipenem; MEM, meropenem; ATM, aztreonam; AMK, amikacin; GEN, gentamicin; TOB, tobramycin; CIP, ciprofloxacin

**Figure S2**

Figure S2. Overall susceptibility of all CF isolates (%), irrespective of source and site of infection (N=199)

C/T, ceftolozane/tazobactam; TZP, piperacillin/tazobactam; CAZ, ceftazidime; IPM, imipenem; MEM, meropenem; ATM, aztreonam; AMK, amikacin; GEN, gentamicin; TOB, tobramycin; CIP, ciprofloxacin
